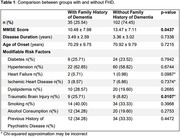# Association between family history of dementia and clinical presentation in dementia patients in Southern Brazil

**DOI:** 10.1002/alz70860_103414

**Published:** 2025-12-23

**Authors:** Henrique Werner Balbinot

**Affiliations:** ^1^ Hospital de Clínicas de Porto Alegre, Porto Alegre, Rio Grande do Sul, Brazil

## Abstract

**Background:**

The Family History of Dementia (FHD) is a well‐known risk factor for developing the disease. This study aims to investigate the association between FHD and the clinical presentation of dementia patients in outpatient clinic in Southern Brazil.

**Method:**

A cross‐sectional study was conducted using a database collected at the dementia outpatient clinic of Hospital de Clínicas de Porto Alegre, involving 137 patients with dementia, who were classified into two groups: with and without FHD. Clinical presentation was evaluated based on the following variables, collected at the patient's first visit: age of onset, disease duration, Mini‐Mental State Examination (MMSE) scores, and the proportion of modifiable risk factors (diabetes, hypertension, heart failure, ischemic heart disease, dyslipidemia, traumatic brain injury, smoking, alcohol consumption, and previous history of psychiatric disease). Data were analyzed using RStudio, applying Mann‐Whitney tests to measure the association of each group with disease duration, age of onset, and MMSE score, and Chi‐square tests to assess the relationship between FHD and modifiable risk factors.

**Result:**

Among the 137 patients (86 women; mean education level of 4.91 ± 4.09 years; 106 white), only 35 (25.54%) had FHD. There was a significant difference in MMSE scores between the two groups (*p* = 0.0437), in which patients with FHD showed lower scores compared to those without FHD (Table 1). However, no significant differences were found for disease duration (*p* = 0.7338), age of onset (*p* = 0.7215), or the proportion of modifiable risk factors (*p*‐values in Table 1), except for “traumatic brain injury” (*p* = 0.0107).

**Conclusion:**

Considering the lower MMSE scores, FHD was associated with a clinical presentation with greater cognitive decline, upon arrival at the outpatient clinic. However, there were no associations with early onset, prolonged duration, or a higher proportion of modifiable risk factors.